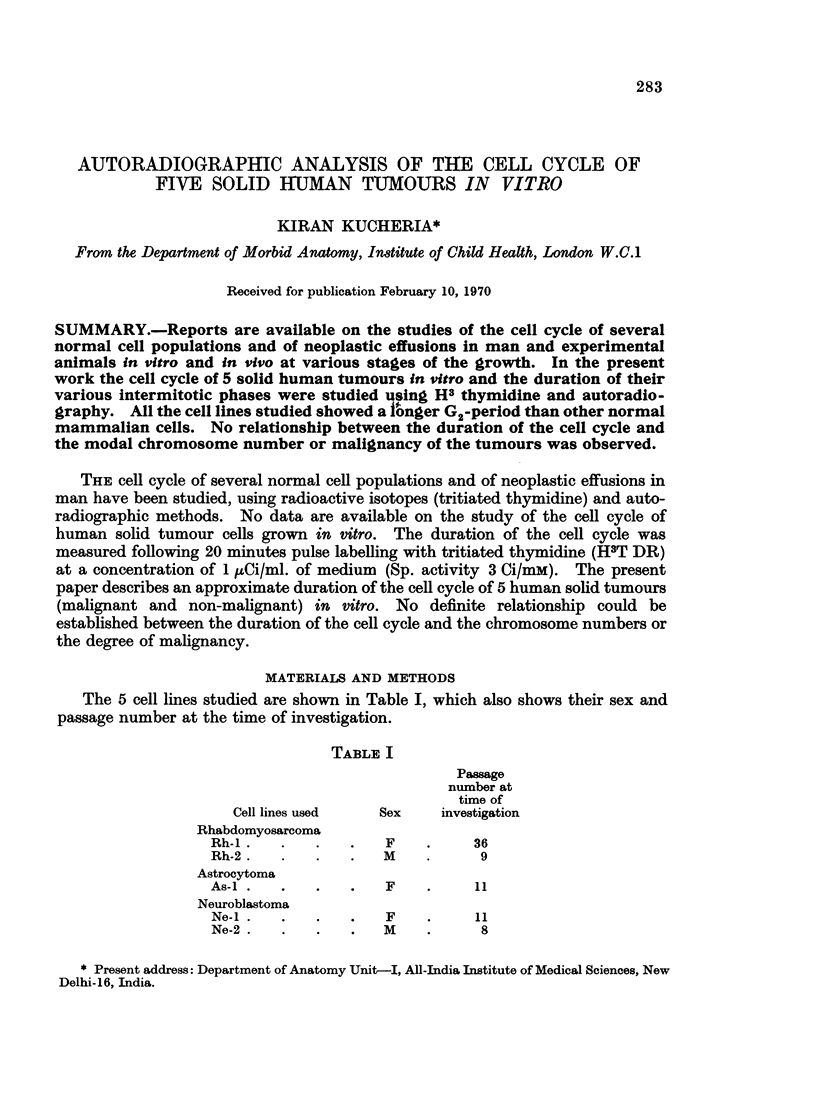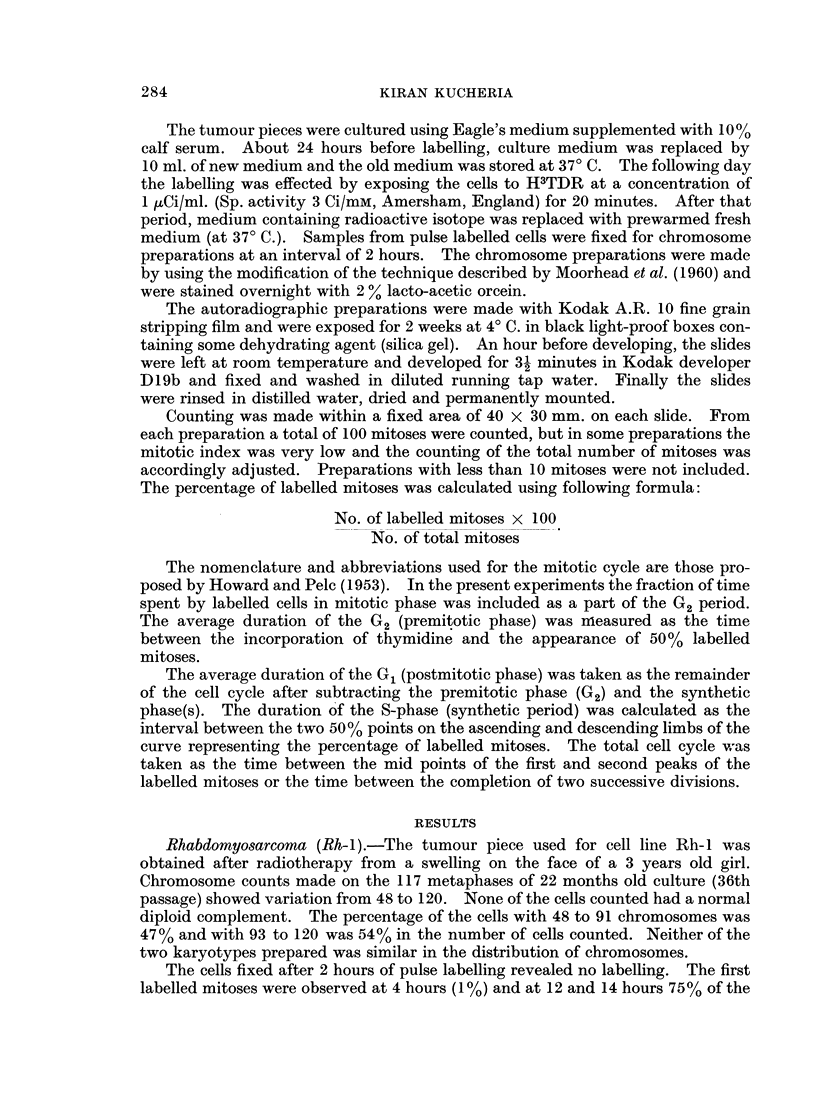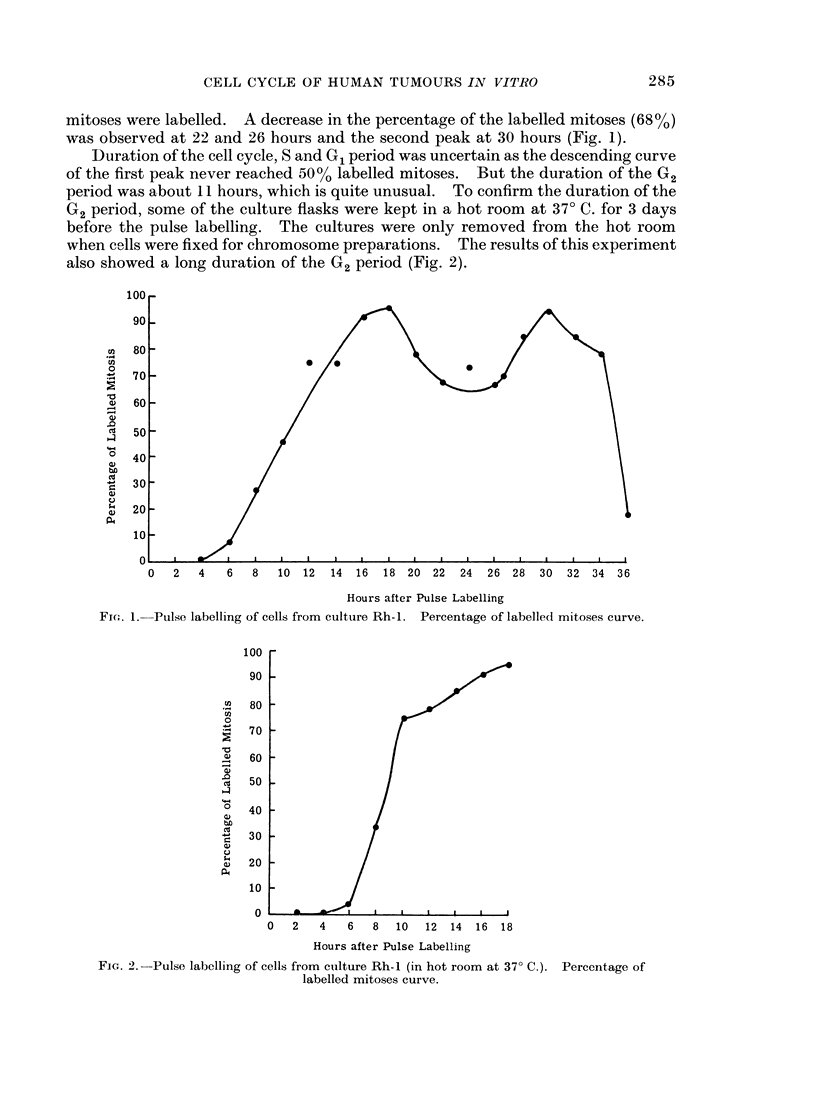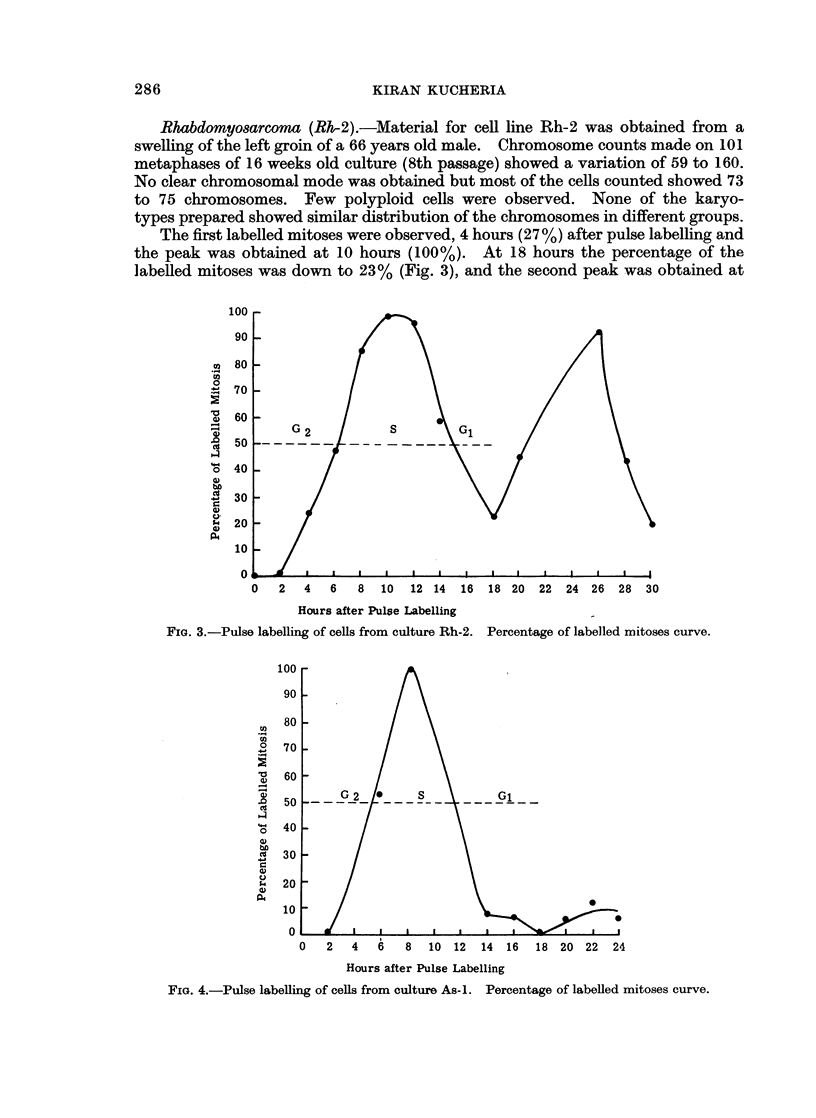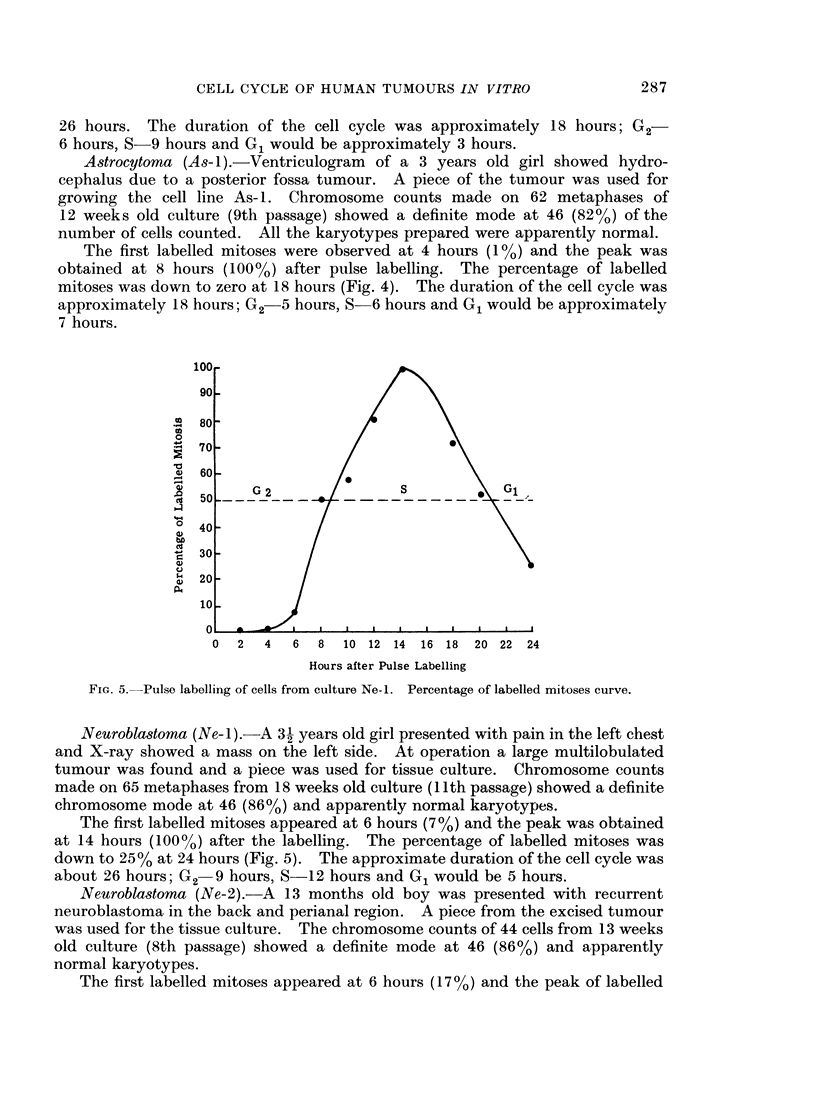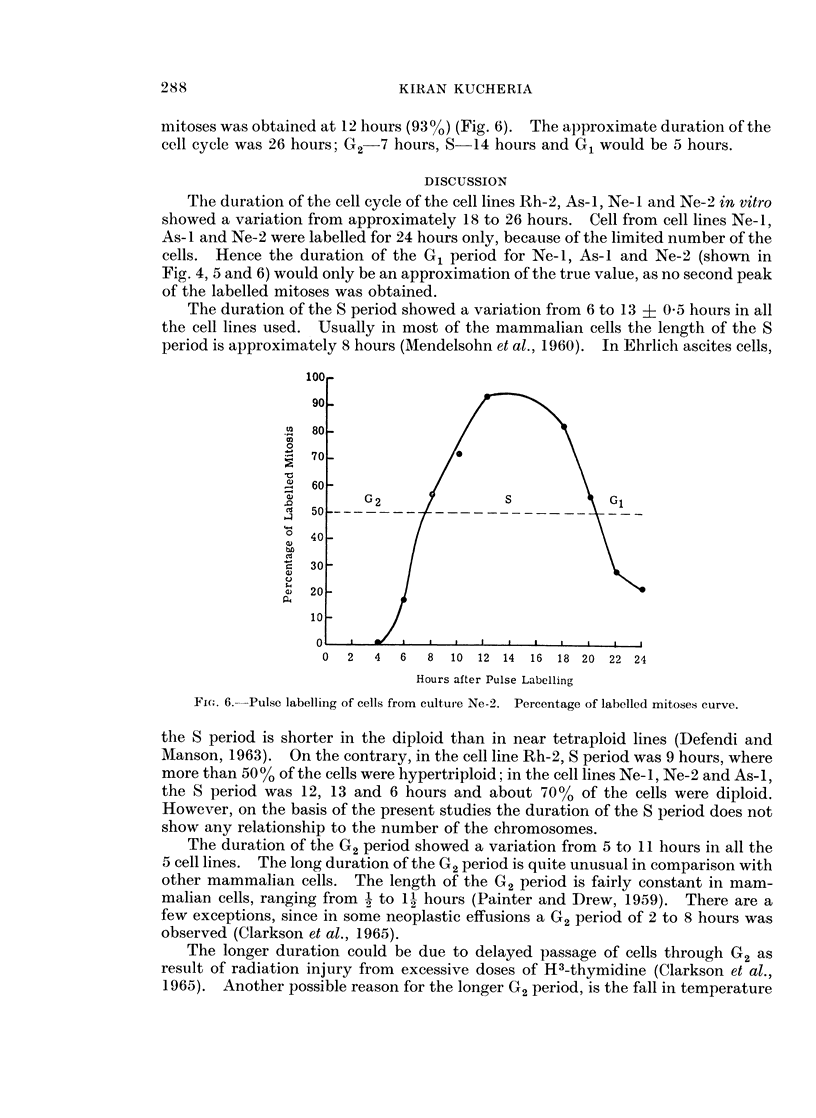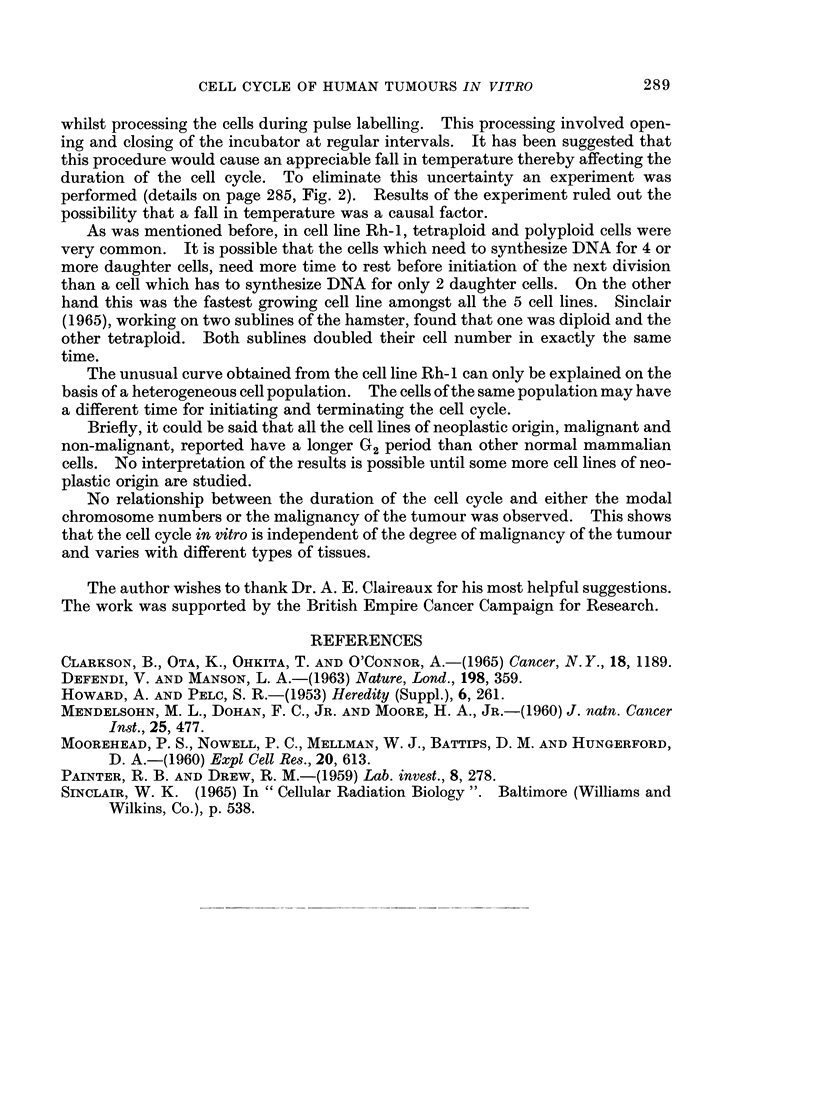# Autoradiographic Analysis of the Cell Cycle of Five Solid Human Tumours In Vitro

**DOI:** 10.1038/bjc.1970.32

**Published:** 1970-06

**Authors:** Kiran Kucheria

## Abstract

Reports are available on the studies of the cell cycle of several normal cell populations and of neoplastic effusions in man and experimental animals *in vitro* and *in vivo* at various stages of the growth. In the present work the cell cycle of 5 solid human tumours *in vitro* and the duration of their various intermitotic phases were studied using H^3^ thymidine and autoradiography. All the cell lines studied showed a longer G_2_-period than other normal mammalian cells. No relationship between the duration of the cell cycle and the modal chromosome number or malignancy of the tumours was observed.


### AUTORADIOGRAPHIC ANALYSIS OF THE CELL CYCLE OF FIVE SOLID HUMAN TUMOURS *IN VITRO*

KIRAN KUCHERIA*

*From the Department of Morbid Anatomy, Institute of Child Health, London W.C.1*

Received for publication February 10, 1970

**SUMMARY.—Reports are available on the studies of the cell cycle of several normal cell populations and of neoplastic effusions in man and experimental animals *in vitro* and *in vivo* at various stages of the growth. In the present work the cell cycle of 5 solid human tumours *in vitro* and the duration of their various intermitotic phases were studied using $H^3$ thymidine and autoradiography. All the cell lines studied showed a longer $G_2$-period than other normal mammalian cells. No relationship between the duration of the cell cycle and the modal chromosome number or malignancy of the tumours was observed.**

THE cell cycle of several normal cell populations and of neoplastic effusions in man have been studied, using radioactive isotopes (tritiated thymidine) and autoradiographic methods. No data are available on the study of the cell cycle of human solid tumour cells grown *in vitro*. The duration of the cell cycle was measured following 20 minutes pulse labelling with tritiated thymidine ($H^3$T DR) at a concentration of 1 $\mu$Ci/ml. of medium (Sp. activity 3 Ci/mM). The present paper describes an approximate duration of the cell cycle of 5 human solid tumours (malignant and non-malignant) *in vitro*. No definite relationship could be established between the duration of the cell cycle and the chromosome numbers or the degree of malignancy.

### MATERIALS AND METHODS

The 5 cell lines studied are shown in Table I, which also shows their sex and passage number at the time of investigation.

TABLE I

| Cell lines used | Sex | Passage number at time of investigation |
|---|---|---|
| Rhabdomyosarcoma | | |
| Rh-1 | F | 36 |
| Rh-2 | M | 9 |
| Astrocytoma | | |
| As-1 | F | 11 |
| Neuroblastoma | | |
| Ne-1 | F | 11 |
| Ne-2 | M | 8 |

* Present address: Department of Anatomy Unit—I, All-India Institute of Medical Sciences, New Delhi-16, India.

The tumour pieces were cultured using Eagle's medium supplemented with 10% calf serum. About 24 hours before labelling, culture medium was replaced by 10 ml. of new medium and the old medium was stored at 37° C. The following day the labelling was effected by exposing the cells to $H^3TDR$ at a concentration of 1 $\mu$Ci/ml. (Sp. activity 3 Ci/mM, Amersham, England) for 20 minutes. After that period, medium containing radioactive isotope was replaced with prewarmed fresh medium (at 37° C.). Samples from pulse labelled cells were fixed for chromosome preparations at an interval of 2 hours. The chromosome preparations were made by using the modification of the technique described by Moorhead *et al.* (1960) and were stained overnight with 2% lacto-acetic orcein.

The autoradiographic preparations were made with Kodak A.R. 10 fine grain stripping film and were exposed for 2 weeks at 4° C. in black light-proof boxes containing some dehydrating agent (silica gel). An hour before developing, the slides were left at room temperature and developed for 3½ minutes in Kodak developer D19b and fixed and washed in diluted running tap water. Finally the slides were rinsed in distilled water, dried and permanently mounted.

Counting was made within a fixed area of 40 × 30 mm. on each slide. From each preparation a total of 100 mitoses were counted, but in some preparations the mitotic index was very low and the counting of the total number of mitoses was accordingly adjusted. Preparations with less than 10 mitoses were not included. The percentage of labelled mitoses was calculated using following formula:

$$\frac{\text{No. of labelled mitoses} \times 100}{\text{No. of total mitoses}}.$$

The nomenclature and abbreviations used for the mitotic cycle are those proposed by Howard and Pelc (1953). In the present experiments the fraction of time spent by labelled cells in mitotic phase was included as a part of the $G_2$ period. The average duration of the $G_2$ (premitotic phase) was measured as the time between the incorporation of thymidine and the appearance of 50% labelled mitoses.

The average duration of the $G_1$ (postmitotic phase) was taken as the remainder of the cell cycle after subtracting the premitotic phase ($G_2$) and the synthetic phase(s). The duration of the S-phase (synthetic period) was calculated as the interval between the two 50% points on the ascending and descending limbs of the curve representing the percentage of labelled mitoses. The total cell cycle was taken as the time between the mid points of the first and second peaks of the labelled mitoses or the time between the completion of two successive divisions.

### RESULTS

*Rhabdomyosarcoma* (*Rh*-1).—The tumour piece used for cell line Rh-1 was obtained after radiotherapy from a swelling on the face of a 3 years old girl. Chromosome counts made on the 117 metaphases of 22 months old culture (36th passage) showed variation from 48 to 120. None of the cells counted had a normal diploid complement. The percentage of the cells with 48 to 91 chromosomes was 47% and with 93 to 120 was 54% in the number of cells counted. Neither of the two karyotypes prepared was similar in the distribution of chromosomes.

The cells fixed after 2 hours of pulse labelling revealed no labelling. The first labelled mitoses were observed at 4 hours (1%) and at 12 and 14 hours 75% of the mitoses were labelled. A decrease in the percentage of the labelled mitoses (68%) was observed at 22 and 26 hours and the second peak at 30 hours (Fig. 1).

Duration of the cell cycle, S and $G_1$ period was uncertain as the descending curve of the first peak never reached 50% labelled mitoses. But the duration of the $G_2$ period was about 11 hours, which is quite unusual. To confirm the duration of the $G_2$ period, some of the culture flasks were kept in a hot room at 37° C. for 3 days before the pulse labelling. The cultures were only removed from the hot room when cells were fixed for chromosome preparations. The results of this experiment also showed a long duration of the $G_2$ period (Fig. 2).

FIG. 1.—Pulse labelling of cells from culture Rh-1. Percentage of labelled mitoses curve.

FIG. 2.—Pulse labelling of cells from culture Rh-1 (in hot room at 37° C.). Percentage of labelled mitoses curve.

*Rhabdomyosarcoma* (*Rh*-2).—Material for cell line Rh-2 was obtained from a swelling of the left groin of a 66 years old male. Chromosome counts made on 101 metaphases of 16 weeks old culture (8th passage) showed a variation of 59 to 160. No clear chromosomal mode was obtained but most of the cells counted showed 73 to 75 chromosomes. Few polyploid cells were observed. None of the karyotypes prepared showed similar distribution of the chromosomes in different groups.

The first labelled mitoses were observed, 4 hours (27%) after pulse labelling and the peak was obtained at 10 hours (100%). At 18 hours the percentage of the labelled mitoses was down to 23% (Fig. 3), and the second peak was obtained at

FIG. 3.—Pulse labelling of cells from culture Rh-2. Percentage of labelled mitoses curve.

FIG. 4.—Pulse labelling of cells from culture As-1. Percentage of labelled mitoses curve.

26 hours. The duration of the cell cycle was approximately 18 hours; $G_2$—6 hours, S—9 hours and $G_1$ would be approximately 3 hours.

*Astrocytoma* (*As*-1).—Ventriculogram of a 3 years old girl showed hydrocephalus due to a posterior fossa tumour. A piece of the tumour was used for growing the cell line As-1. Chromosome counts made on 62 metaphases of 12 weeks old culture (9th passage) showed a definite mode at 46 (82%) of the number of cells counted. All the karyotypes prepared were apparently normal.

The first labelled mitoses were observed at 4 hours (1%) and the peak was obtained at 8 hours (100%) after pulse labelling. The percentage of labelled mitoses was down to zero at 18 hours (Fig. 4). The duration of the cell cycle was approximately 18 hours; $G_2$—5 hours, S—6 hours and $G_1$ would be approximately 7 hours.

FIG. 5.—Pulse labelling of cells from culture Ne-1. Percentage of labelled mitoses curve.

*Neuroblastoma* (*Ne*-1).—A 3½ years old girl presented with pain in the left chest and X-ray showed a mass on the left side. At operation a large multilobulated tumour was found and a piece was used for tissue culture. Chromosome counts made on 65 metaphases from 18 weeks old culture (11th passage) showed a definite chromosome mode at 46 (86%) and apparently normal karyotypes.

The first labelled mitoses appeared at 6 hours (7%) and the peak was obtained at 14 hours (100%) after the labelling. The percentage of labelled mitoses was down to 25% at 24 hours (Fig. 5). The approximate duration of the cell cycle was about 26 hours; $G_2$—9 hours, S—12 hours and $G_1$ would be 5 hours.

*Neuroblastoma* (*Ne*-2).—A 13 months old boy was presented with recurrent neuroblastoma in the back and perianal region. A piece from the excised tumour was used for the tissue culture. The chromosome counts of 44 cells from 13 weeks old culture (8th passage) showed a definite mode at 46 (86%) and apparently normal karyotypes.

The first labelled mitoses appeared at 6 hours (17%) and the peak of labelled mitoses was obtained at 12 hours (93%) (Fig. 6). The approximate duration of the cell cycle was 26 hours; $G_2$—7 hours, S—14 hours and $G_1$ would be 5 hours.

### DISCUSSION

The duration of the cell cycle of the cell lines Rh-2, As-1, Ne-1 and Ne-2 *in vitro* showed a variation from approximately 18 to 26 hours. Cell from cell lines Ne-1, As-1 and Ne-2 were labelled for 24 hours only, because of the limited number of the cells. Hence the duration of the $G_1$ period for Ne-1, As-1 and Ne-2 (shown in Fig. 4, 5 and 6) would only be an approximation of the true value, as no second peak of the labelled mitoses was obtained.

The duration of the S period showed a variation from 6 to 13 ± 0·5 hours in all the cell lines used. Usually in most of the mammalian cells the length of the S period is approximately 8 hours (Mendelsohn *et al.*, 1960). In Ehrlich ascites cells,

FIG. 6.—Pulse labelling of cells from culture Ne-2. Percentage of labelled mitoses curve.

the S period is shorter in the diploid than in near tetraploid lines (Defendi and Manson, 1963). On the contrary, in the cell line Rh-2, S period was 9 hours, where more than 50% of the cells were hypertriploid; in the cell lines Ne-1, Ne-2 and As-1, the S period was 12, 13 and 6 hours and about 70% of the cells were diploid. However, on the basis of the present studies the duration of the S period does not show any relationship to the number of the chromosomes.

The duration of the $G_2$ period showed a variation from 5 to 11 hours in all the 5 cell lines. The long duration of the $G_2$ period is quite unusual in comparison with other mammalian cells. The length of the $G_2$ period is fairly constant in mammalian cells, ranging from $\frac{1}{2}$ to $1\frac{1}{2}$ hours (Painter and Drew, 1959). There are a few exceptions, since in some neoplastic effusions a $G_2$ period of 2 to 8 hours was observed (Clarkson *et al.*, 1965).

The longer duration could be due to delayed passage of cells through $G_2$ as result of radiation injury from excessive doses of $H^3$-thymidine (Clarkson *et al.*, 1965). Another possible reason for the longer $G_2$ period, is the fall in temperature whilst processing the cells during pulse labelling. This processing involved opening and closing of the incubator at regular intervals. It has been suggested that this procedure would cause an appreciable fall in temperature thereby affecting the duration of the cell cycle. To eliminate this uncertainty an experiment was performed (details on page 285, Fig. 2). Results of the experiment ruled out the possibility that a fall in temperature was a causal factor.

As was mentioned before, in cell line Rh-1, tetraploid and polyploid cells were very common. It is possible that the cells which need to synthesize DNA for 4 or more daughter cells, need more time to rest before initiation of the next division than a cell which has to synthesize DNA for only 2 daughter cells. On the other hand this was the fastest growing cell line amongst all the 5 cell lines. Sinclair (1965), working on two sublines of the hamster, found that one was diploid and the other tetraploid. Both sublines doubled their cell number in exactly the same time.

The unusual curve obtained from the cell line Rh-1 can only be explained on the basis of a heterogeneous cell population. The cells of the same population may have a different time for initiating and terminating the cell cycle.

Briefly, it could be said that all the cell lines of neoplastic origin, malignant and non-malignant, reported have a longer $G_2$ period than other normal mammalian cells. No interpretation of the results is possible until some more cell lines of neoplastic origin are studied.

No relationship between the duration of the cell cycle and either the modal chromosome numbers or the malignancy of the tumour was observed. This shows that the cell cycle *in vitro* is independent of the degree of malignancy of the tumour and varies with different types of tissues.

The author wishes to thank Dr. A. E. Claireaux for his most helpful suggestions. The work was supported by the British Empire Cancer Campaign for Research.

### REFERENCES

CLARKSON, B., OTA, K., OHKITA, T. AND O'CONNOR, A.—(1965) *Cancer, N.Y.*, **18**, 1189.
DEFENDI, V. AND MANSON, L. A.—(1963) *Nature, Lond.*, **198**, 359.
HOWARD, A. AND PELC, S. R.—(1953) *Heredity* (Suppl.), **6**, 261.
MENDELSOHN, M. L., DOHAN, F. C., JR. AND MOORE, H. A., JR.—(1960) *J. natn. Cancer Inst.*, **25**, 477.
MOOREHEAD, P. S., NOWELL, P. C., MELLMAN, W. J., BATTIPS, D. M. AND HUNGERFORD, D. A.—(1960) *Expl Cell Res.*, **20**, 613.
PAINTER, R. B. AND DREW, R. M.—(1959) *Lab. invest.*, **8**, 278.
SINCLAIR, W. K. (1965) In " Cellular Radiation Biology ". Baltimore (Williams and Wilkins, Co.), p. 538.